# A dual-moderation model of physical activity and gender in the relationship between experiential avoidance and short video dependency

**DOI:** 10.3389/fpsyg.2026.1757041

**Published:** 2026-08-31

**Authors:** Jian Lei, Xinxin Tan

**Affiliations:** 1 Hunan Institute of Science and Engineering, Yongzhou, China; 2 Zhangjiajie College, Zhangjiajie, China; 3 Jishou University, Jishou, China

**Keywords:** adolescent, experiential avoidance, gender, physical activity, short video dependency

## Abstract

The rapid proliferation of short video platforms has raised concerns about adolescent media dependency. This study examined how experiential avoidance (EA) relates to short video dependency (SVD), considering the moderating effects of physical activity (PA) and gender. A total of 3,964 adolescents from four Chinese provinces completed self-report questionnaires on EA, short video use, and PA. Moderation analyses using SPSS PROCESS showed that EA was positively associated with SVD. PA significantly attenuated this relationship, suggesting a protective role, and this association was further moderated by gender, with girls showing a stronger EA-SVD association than boys. Simple slope analyses indicated that the buffering role of PA was most pronounced among girls with high EA. These findings highlight the complex interplay of individual behavioral habits and gender in shaping adolescent SVD. The findings suggest practical ways to reduce SVD, such as promoting PA and applying strategies that consider gender differences.

## 1 Introduction

### 1.1 Short video dependency

In recent years, the rapid development of mobile internet technologies and artificial intelligence algorithms has propelled short videos to the forefront of information dissemination and entertainment consumption. Compared with traditional media, short videos—driven by algorithmic recommendation, low-barrier content creation, and real-time interactive feedback—offer heightened entertainment value and immersion. These features have led to their explosive popularity and global user engagement. TikTok, as the leading platform, had reached 1.677 billion global users and accumulated more than 3.5 billion downloads by the end of 2023, representing a 79.1% increase since 2020 (Chen et al., 2020). In China, the trend is equally striking: The statistics of 1.067 billion online video users and 1.053 billion short-video users are from the 53rd Statistical Report on China’s Internet Development issued by CNNIC. While this surge has fueled the growth of new media industries, it has also generated emerging psychosocial challenges, particularly short video dependency (SVD). To maintain a clear focus on short video dependency rather than general Internet or social media addiction, the present study emphasizes the platform-specific features of short videos, including algorithmic recommendation, rapid feedback, fragmented content, instant gratification, and immersive scrolling. These characteristics make short video platforms especially attractive to adolescents who seek quick emotional relief or distraction from unpleasant internal experiences. SVD is typically defined as a persistent and intense craving for short videos, coupled with impaired self-control (Chung, 2022). This condition manifests in multiple domains of functional impairment, including difficulties in time management, reduced academic and work efficiency, heightened anxiety and depression, and increased social withdrawal. Recent review studies have indicated that SVD constitutes a complex behavioral problem shaped by the joint influence of individual psychological vulnerabilities, social environmental factors, and platform-specific algorithmic and content characteristics. At its core, this dependency is underpinned by deficits in emotion regulation and maladaptive coping strategies, which increase individuals’ susceptibility to excessive and compulsive short video use (Liao, 2024; Xiong et al., 2024). From a theoretical perspective, the emotion regulation model helps explain this behavior. According to the model, individuals facing negative emotions such as stress, shame, or failure may turn to media use to escape internal conflicts and gain temporary relief (Shen et al., 2025; Wang J. et al., 2024). Short videos, with their instant gratification, content diversity, and immersive experiences, serve as an especially effective tool for such escapist use (Segerstrom and Smith, 2019). However, over time, this compensatory use may evolve into dependency, resulting in more severe psychological and behavioral problems. Empirical studies have demonstrated that SVD undermines attention and executive functioning, thereby impairing academic and occupational performance (Dong et al., 2023). It is also strongly associated with loneliness, depression, sleep disturbances (Qin et al., 2022), and diminished real-world social competence and self-development potential (Cardoso-Leite and Bavelier, 2014). Adolescents are widely recognized as a high-risk group for developing SVD, as their cognitive control systems remain immature, their emotion regulation mechanisms are relatively fragile, and their sensitivity to external stimuli is heightened (Zhang et al., 2023). Indeed, estimates indicate that about 22 to 32% of adolescents exhibit problematic short video use (Liu et al., 2021). The adverse consequences of this dependency may not only hinder academic and social functioning in the short term but may also exert long-lasting effects on psychological development trajectories. Excessive reliance on short videos during this critical developmental stage can profoundly affect mental health, socialization processes, and personality formation. Against this backdrop, a systematic investigation of the factors influencing adolescent SVD is both theoretically and practically significant. Importantly, this study does not aim to explore mediating mechanisms, but instead focuses on moderating effects. While mediation highlights how or why relationships occur, moderation clarifies the conditions under which these relationships vary. Building on this perspective, the present study examines the moderating roles of PA and gender in the relationship between EA and SVD. By doing so, it aims to elucidate how behavioral habits and demographic characteristics shape the development of media dependency, thereby offering guidance for more precise interventions and group-specific prevention strategies.

### 1.2 Experiential avoidance and short video dependency

Experiential avoidance (EA) refers to the psychological tendency to deliberately evade, suppress, or alter unpleasant internal experiences for the purpose of self-regulation (Hayes, 1994). Although closely related to concepts such as emotion regulation and avoidant coping, EA is theoretically and functionally distinct. Emotion regulation is a broader construct encompassing diverse strategies individuals employ to influence the experience and expression of emotions, including both adaptive strategies (e.g., cognitive reappraisal) and maladaptive strategies (e.g., suppression and avoidance) (Gross, 2015). Avoidant coping, by contrast, emphasizes behavioral avoidance of external stressors, such as denial, withdrawal, or distraction (Melodia et al., 2022). Escapism, in contrast, primarily refers to individuals’ motivation to temporarily disengage from real-life stressors by immersing themselves in external activities, such as online entertainment or short video consumption. Its conceptual focus lies in usage motives and behavioral purposes rather than internal experiential processes. Using network analysis, Hong et al. (2025) revealed complex interactions between individual psychological characteristics—including escapist motivation—and platform-specific features of short video applications. Furthermore, a three-wave longitudinal study by Jouhki et al. (2022) demonstrated that escapism is a robust psychological predictor of excessive online behaviors, providing strong longitudinal evidence for its role in the development of SVD. Epidemiological data indicate that about half of individuals show moderate to high EA (Akbari et al., 2022). Common behavioral manifestations include avoiding conflict, suppressing emotions, and resisting self-disclosure (Berman et al., 2010). Persistent reliance on this strategy undermines psychological resilience and is considered a critical antecedent of maladaptive behaviors and psychological distress. In the context of digital media proliferation, short video platforms—with their instant feedback, immersive experiences, and high accessibility—offer adolescents a convenient means of escaping real-life difficulties (Yang and Liu, 2025; Yi et al., 2025a). The Compensatory Internet Use Model posits that when individuals face dissatisfaction or stress in daily life, online activities may become an alternative source of gratification (Kardefelt-Winther, 2014a). Adolescents, in particular, may immerse themselves in the pleasurable stimulation of short videos to avoid academic pressure, family conflicts, or peer-related tensions (Yang et al., 2022). From the perspective of Self-Regulation Failure Theory (Baumeister and Heatherton, 1996), when individuals experience limited emotional resources or impaired self-control, they are more likely to rely on external, immediate rewards for short-term emotional relief. The low entry threshold and high-frequency reward mechanisms of short video platforms make them particularly well-suited to fulfilling the psychological needs of individuals high in EA, thereby fostering dependency. Empirical findings support this theoretical assumption. For example, a large-scale study of 1,591 Chinese students demonstrated that EA significantly and positively predicts levels of SVD (Wang et al., 2025b). This evidence not only highlights EA as a major risk factor for SVD but also underscores its profound implications for adolescent psychological development and media use patterns.

### 1.3 Physical activity as a moderator

Physical activity (PA) refers to bodily movement produced by skeletal muscles that leads to energy expenditure. It includes diverse forms, ranging from daily activities such as walking and household tasks to structured exercise (Caspersen et al., 1985). The World Health Organization (WHO) recommends that adolescents engage in at least 60 min of moderate-to-vigorous PA daily to maintain physiological health, promote psychological balance, and strengthen social adaptability (Bull et al., 2020). In the digital era, PA is widely regarded as a protective factor against online dependency among adolescents (Gan et al., 2025; Liu et al., 2024b; Liu et al., 2025a; Liu et al., 2024d; Liu et al., 2025b; Wang et al., 2025c; Yan et al., 2026b). The Conservation of Resources (COR) Theory provides a framework for understanding this protective effect. It suggests that individuals rely on internal and external resources to maintain psychological stability under stress, and that losing resources can heighten negative emotions and lead to maladaptive coping (Hobfoll, 1989). PA replenishes physiological resources by stimulating beneficial neurobiological responses, such as increased secretion of β-endorphins, serotonin, and dopamine. At the same time, it strengthens psychological resources by enhancing attentional control, cognitive flexibility, and emotional regulation (Jia et al., 2025; Liu et al., 2024a,c; Peng et al., 2025a; Yan et al., 2026a; Yang et al., 2025, 2026; Yi et al., 2025b). Together, these effects reduce the need for compensatory reliance on digital media such as short video platforms. Recent research has shown that the protective effect of PA on short video addiction operates through chained pathways linked to self-regulation–related psychological factors, supporting its indirect role in addictive media use (Peng B. et al., 2025; Peng et al., 2025a,b). Similarly, Jianfeng et al. (2024) found that higher levels of PA are associated with a lower likelihood of short video addiction in adolescents. Furthermore, Wu et al. found that autonomous motivation and mental health literacy mediate this protective effect, acting as key psychological pathways through which PA lowers the risk of addiction (Wu et al., 2026). For instance, survey data from Chinese adolescents demonstrate that regular PA is not only negatively associated with short video addiction but also predicts lower levels of problematic internet use (Ferguson et al., 2023). Within the relationship between EA and SVD, PA may serve as a moderating factor. Adolescents with high EA are prone to using short videos for immediate entertainment and immersion to escape negative experiences such as loneliness, anxiety, and helplessness. Over time, this coping strategy may evolve into dependency. However, PA has been consistently shown to alleviate anxiety, depression, and stress (Liu et al., 2024d; Luo et al., 2025; Wang W. et al., 2025; Yin et al., 2025). As such, it may functionally substitute for the emotional buffering role of short videos, thereby weakening the association between EA and dependency. Cross-cultural evidence further corroborates this moderating role. For example, a United States study found that high levels of PA significantly weakened the positive association between avoidant coping and internet addiction, whereas this link was considerably stronger among adolescents with low PA (Chou et al., 2018). These findings suggest that PA not only exerts a direct negative association with SVD but also modifies the strength of EA pathways. Two mechanisms may explain this moderating effect. First, as an alternative emotion regulation strategy, PA provides individuals high in EA with a healthier outlet for releasing negative affect, thereby reducing their reliance on short videos. Second, by strengthening physiological and psychological resources, PA improves self-control and the ability to delay gratification, making adolescents more resistant to the instant rewards offered by short videos and lowering their risk of developing addictive use patterns. Nevertheless, some studies report inconsistent findings regarding the protective effect of PA (Peluso and Guerra, 2005). Factors such as gender, type of exercise, level of social support, and cultural context may influence its moderating function, leading to varying or even nonsignificant effects across different samples. These inconsistencies highlight the complexity and context-dependence of PA as a moderator and suggest the need for replication in larger and more diverse samples (Zhang et al., 2024). Overall, existing theoretical frameworks together with empirical evidence consistently support a moderating role of PA in the relationship between EA and SVD, while indicating that the underlying processes are not straightforward.

### 1.4 Gender as a moderator

Gender differences play an important role in patterns of digital media use and related psychological processes, and may function as a moderator in the association between EA and SVD (Zhang et al., 2022). Both theoretical and empirical studies suggest that females are more likely to adopt emotion-oriented coping strategies when confronted with negative emotions, including avoidance, suppression, and attentional distraction. Consequently, when experiencing loneliness, anxiety, or frustration, females are more prone to rely on short videos and similar media for immediate emotional relief, which increases their risk of developing dependency. By contrast, males tend to use digital media in a more instrumental manner, such as for information seeking, task-oriented activities, or functional operations, which lowers their overall risk of addiction (Tamres et al., 2002). Cross-cultural empirical evidence further supports this gendered pattern. A meta-analysis involving 82,440 participants across 21 countries and regions found that males were more susceptible to developing internet gaming disorder, whereas females exhibited significantly higher levels of dependency on social media and short video platforms (Su et al., 2020). Moreover, prior research shows that the effect of EA on SVD tends to be stronger among females, indicating that gender differences may influence how EA relates to media addiction (Hodges, 2021).

Incorporating gender into the analytical framework thus provides a more precise identification of high-risk populations and offers a theoretical foundation for gender-sensitive intervention strategies. However, inconsistencies remain in the literature. Some studies report that males also display elevated risks of short video or internet-related addictions, which contradicts the notion that females are more vulnerable (Mari et al., 2023). Such discrepancies may stem from variations in sample age, cultural context, media type, measurement tools, or social role expectations. These findings highlight the contextual dependence and complexity of gender as a moderator in the relationship between EA and SVD. Taken together, both theory and evidence suggest that gender not only shapes the choice of emotional regulation strategies but also alters the strength of EA’s effect on SVD, serving as a critical boundary condition.

### 1.5 Current study

**Figure 1:**
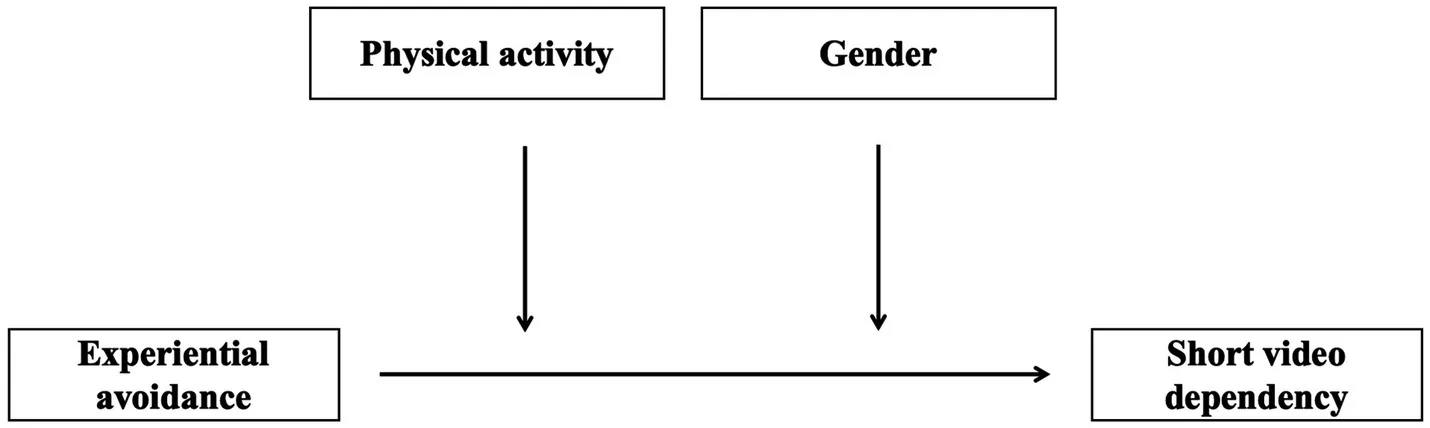
Hypothesized moderation model.

Although prior research has examined the influence of EA on internet addiction, social media use, and other forms of digital behavior, studies focusing specifically on adolescent SVD—a rapidly emerging phenomenon—remain limited. Furthermore, most existing studies treat PA or gender as single moderating variables, without systematically exploring their combined moderating effects. This gap restricts a comprehensive understanding of adolescent media use and its psychological mechanisms. To address this gap, the present study adopts a dual moderation perspective to investigate the relationship between EA and adolescent SVD. Specifically, we test whether PA and gender moderate the predictive effect of EA on SVD, and we analyze the boundary conditions and strength of these moderating effects. By constructing a moderation model (Figure 1), this study aims to determine whether EA exerts differential impacts on SVD across varying levels of PA and between genders.

The contributions of this study are threefold. First, it extends the literature by linking EA with adolescent SVD, thereby providing empirical support for the psychological mechanisms underlying short video use. Second, it incorporates the joint moderating effects of PA and gender, offering a nuanced perspective on individual differences. Third, by examining these moderation pathways, the study provides both theoretical and practical implications for the design of interventions tailored to gender and behavioral patterns.

Taken together, the theoretical logic of the present study is that EA may increase adolescents’ tendency to escape or suppress negative internal experiences through highly accessible short videos, thereby increasing the risk of SVD. PA may provide a healthier coping and self-regulatory resource that weakens this positive association, whereas gender may shape the strength of this pathway because boys and girls may differ in emotional coping preferences and patterns of short video use. On this basis, the present study tested the following directional hypotheses:

*H1*: EA is positively associated with adolescent SVD.

*H2*: PA weakens the positive association between EA and adolescent SVD.

*H3*: Gender moderates the relationship between EA and adolescent SVD, such that the positive association is stronger among girls than boys.

## 2 Methods

### 2.1 Participants

Beginning in November 2025, this study employed a stratified cluster sampling approach to collect data from 4,125 adolescents in Hunan, Sichuan, Guangdong, and Zhejiang provinces, China. Schools were selected from the four provinces, and intact classes within participating schools were invited to complete the survey with the assistance of homeroom teachers. Prior to data collection, the research team contacted teachers at participating schools to explain the study objectives and data usage, and formal approval was obtained from the schools. Paper-based questionnaires were distributed, completed, and collected on site under standardized instructions, and each questionnaire required approximately 15 min to complete. The study was conducted following the Declaration of Helsinki. Researchers explained the study purpose, confidentiality procedures, voluntary nature of participation, and the importance of honest responses. Before distributing the questionnaires, the research team obtained written informed consent from the adolescents themselves and from their parents or legal guardians. Only participants with full consent took part in the study. Data validity was checked before analysis. Responses showing mechanical patterns (e.g., selecting the same option for all items), clear logical inconsistencies (e.g., indicating “rarely use short videos” while simultaneously reporting “often stay up late watching short videos”), or substantial missing/invalid information were excluded. After data cleaning, 161 questionnaires were excluded, yielding a final valid sample of 3,964 adolescents and a valid response rate of 96.10%. The final sample included 1,991 boys and 1,973 girls, consisting of 152 elementary school students, 1,777 middle school students, and 2,035 high school students. The mean age was 14.72 years (SD = 1.44).

### 2.2 Measures

#### 2.2.1 Short video dependency

SVD was measured using the Problematic Short Video Use Scale revised by Mao and Jiang (2023). This 13-item scale assesses three dimensions: cognitive-behavioral changes, physiological impairment, and social engagement. Each item was rated on a 5-point Likert scale (1 = strongly disagree, 5 = strongly agree). Total scores ranged from 13 to 65, with higher scores reflecting greater short video dependency. Cognitive-behavioral changes capture reduced engagement with the external environment (e.g., “Excessive use of short videos makes me indifferent to the outside world and reduces my interaction with others”). Physiological impairment reflects health consequences of excessive use (e.g., “Watching short videos makes me sit for long periods, lack exercise, and often feel tired”). Social adhesion reflects the role of short videos in building or maintaining social connections (e.g., “I recommend short videos I like or find interesting to my friends”). In this study, the scale demonstrated good internal consistency (Cronbach’s *α* = 0.852).

#### 2.2.2 Experiential avoidance

EA was assessed using the Chinese version of the Acceptance and Action Questionnaire II (AAQ-II) (Cao et al., 2013), adapted by Cao Jing and colleagues from the original scale developed by Fledderus et al. (2012). The AAQ-II consists of seven items rated on a 7-point Likert scale (1 = never, 7 = always), with total scores ranging from 7 to 49. Higher scores indicate greater levels of EA and lower psychological flexibility. Although some items (e.g., AAQ3, AAQ6, AAQ7) may not directly measure EA, the overall scale has shown strong psychometric properties, including internal consistency (Cronbach’s *α* = 0.916) and construct validity. The Chinese version has been well validated, is culturally appropriate, and works well for large-scale adolescent surveys, allowing comparison with previous studies (Wang et al., 2025a).

#### 2.2.3 Physical activity

PA was assessed using the PA Rating Scale revised by Liang and Liu (1994). The scale consists of three items assessing exercise intensity, duration, and frequency, with each item rated on a 5-point Likert scale. A composite PA score was calculated using the formula PA = intensity × (duration − 1) × frequency. Thus, this measure should be understood as a composite behavioral index based on intensity, duration, and frequency rather than a traditional multi-item psychological scale. Higher scores indicate higher overall levels of PA. This scoring method has been commonly used in research on adolescent PA in China (Peng et al., 2025b). In this study, the scale showed acceptable reliability for a brief three-item behavioral index (Cronbach’s *α* = 0.623), although this coefficient should be interpreted cautiously because the measure combines different components of activity behavior.

### 2.3 Covariate variables

Age and grade were controlled for, considering participants’ developmental stage and school level. Adolescence is a critical developmental stage, and previous research has consistently shown that age-related maturation and school grade differences are closely associated with adolescents’ media use patterns and self-regulatory abilities. Given the strong correspondence between age and grade within the Chinese educational system, controlling for these variables allowed us to account for basic developmental and schooling-stage effects while maintaining model parsimony.

### 2.4 Data processing and analysis

All questionnaire data were first entered into Excel 2021 and then analyzed using SPSS 26.0. The primary variables included EA, SVD, PA, and gender. Descriptive statistics were computed using means (*M*) and standard deviations (SD), reported as *M* ± SD to provide an intuitive overview of variable distributions. To assess potential common method bias (CMB), Harman’s single-factor test was conducted by including all measurement items of the core variables in an unrotated exploratory factor analysis. Pearson correlation analyses were then conducted to examine the bivariate associations among the key variables, providing preliminary evidence for subsequent moderation analyses. To enhance comparability and stability, all continuous variables were standardized prior to hypothesis testing.

Moderation analyses were conducted using the SPSS PROCESS macro (Model 2) (Hayes, 2018). This approach tested the predictive effect of EA on SVD and examined whether PA and gender moderated this relationship. Because the primary aim of this study was to examine whether PA and gender independently moderated the association between EA and SVD, rather than to test their joint interactive moderating effect, PROCESS Model 2 was selected in accordance with the theoretical hypotheses and model specification. Accordingly, the analysis focused on the two-way interaction terms (EA × PA and EA × gender), without estimating a three-way interaction term. Model parameters and their 95% confidence intervals (95% CI) were estimated using 5,000 bootstrap resamples to ensure robustness and reliability (Berkovits et al., 2000). Age and grade were treated as covariates to account for possible confounding effects. The threshold for statistical significance was set at *α* = 0.05. Moderation effects were considered significant if the bootstrapped 95% CI did not include zero. These analytic strategies were designed to ensure accuracy and rigor in testing the relationship between EA and adolescent SVD, as well as the boundary conditions under which this association may vary.

## 3 Results

### 3.1 Test for common method bias

Harman’s single-factor test was conducted by including all measurement items of the core variables in an unrotated exploratory factor analysis. The analysis identified three factors with eigenvalues above one, and the first factor explained 31.63% of the variance, which is below the commonly used 50% threshold. These results suggest that common method bias was unlikely to seriously affect the study findings.

### 3.2 Descriptive statistics

The results presented in Table 1 indicate significant gender differences in PA (*t* = 9.03, *p* < 0.001), EA (*t* = −4.78, *p* < 0.001), and SVD (*t* = −4.37, *p* < 0.001). Specifically, boys reported higher levels of PA, whereas girls scored higher on EA and SVD.

**Table 1:** Descriptive statistics by gender and school stage.

Variables	PA		EA		SVD	
	Mean	SD	Mean	SD	Mean	SD
Boys	19.38	22.39	21.04	10.30	36.14	10.81
Girls	14.18	16.64	22.58	9.94	37.63	10.65
*t*	9.03***		−4.78***		−4.37***	
Primary school	14.65	21.29	21.65	9.81	36.95	11.04
Junior high school	19.62	19.46	20.39	10.26	35.42	10.90
Senior high school	14.92	20.00	23.05	9.92	38.14	10.45
*F*	27.826***		33.270***		30.679***	

PA, physical activity; EA, experiential avoidance; SVD, short video dependency. ***p < 0.001.

Significant grade-level differences were also observed in PA (*F* = 27.826, *p* < 0.001), EA (*F* = 33.270, *p* < 0.001), and SVD (*F* = 30.679, *p* < 0.001). Adolescents in middle school reported higher PA compared with those in elementary or high school. Conversely, high school students demonstrated the highest levels of EA and SVD, followed by elementary school students, with middle school students scoring lowest.

### 3.3 Correlation analysis

As shown in Table 2, EA was significantly negatively correlated with PA (*r* = −0.106, *p* < 0.001) and significantly positively correlated with SVD (*r* = 0.439, *p* < 0.001). In addition, SVD was significantly negatively correlated with PA (*r* = −0.090, *p* < 0.001). These correlations provided preliminary evidence supporting the hypothesized relationships among the variables.

**Table 2:** Correlations among main variables.

Variables	1	2	3
1 EA	—		
2 PA	−0.106***	—	
3 SVD	0.439***	−0.090***	—

EA, experiential avoidance; PA, physical activity; SVD, short video dependency; ***p < 0.001.

### 3.4 Moderation model testing

**Figure 2:**
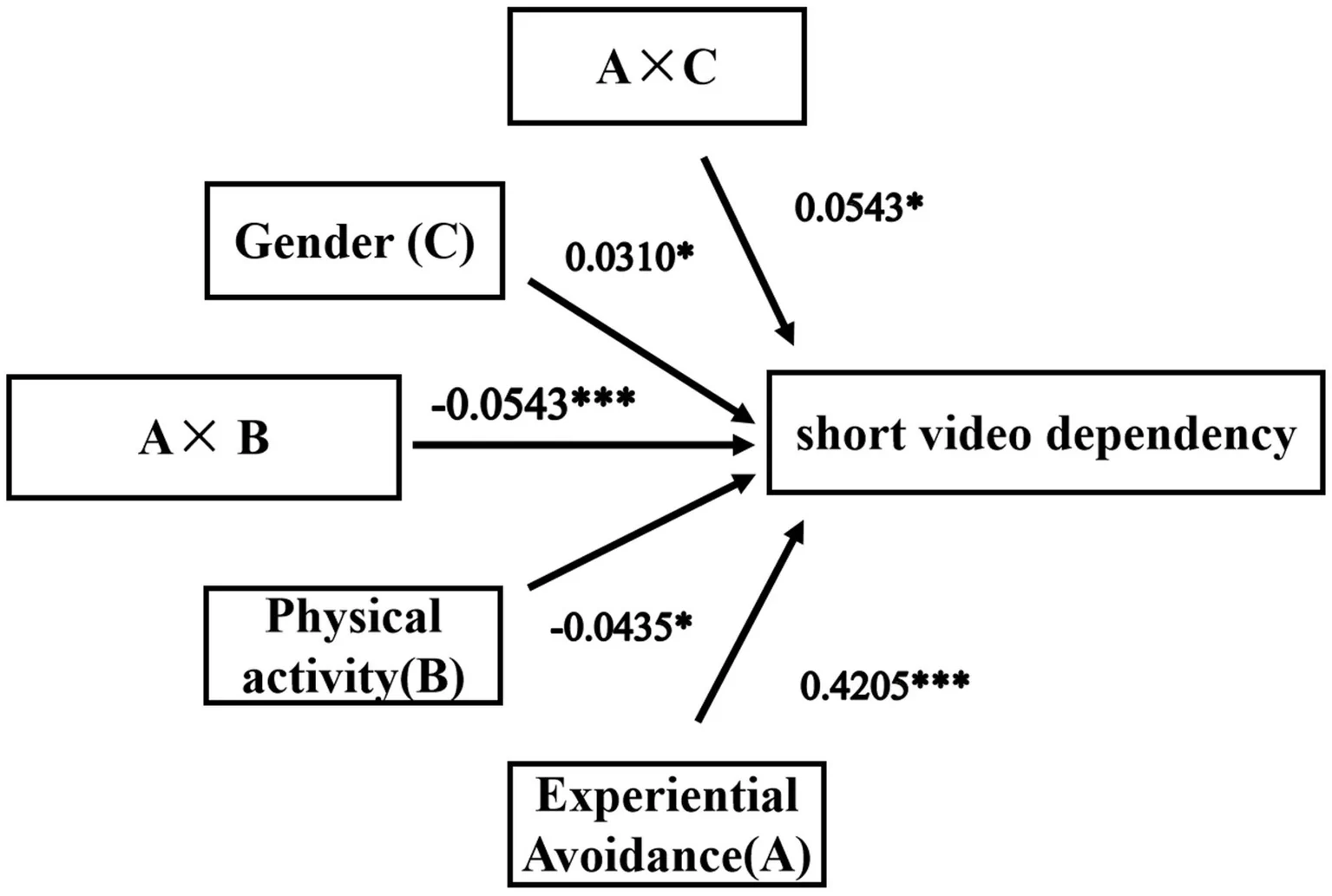
Statistical moderation model (*p < 0.05; ***p < 0.001). EA, experiential avoidance; PA, physical activity; SVD, short video dependency; gender was coded as boys = 0 and girls = 1.

**Figure 3:**
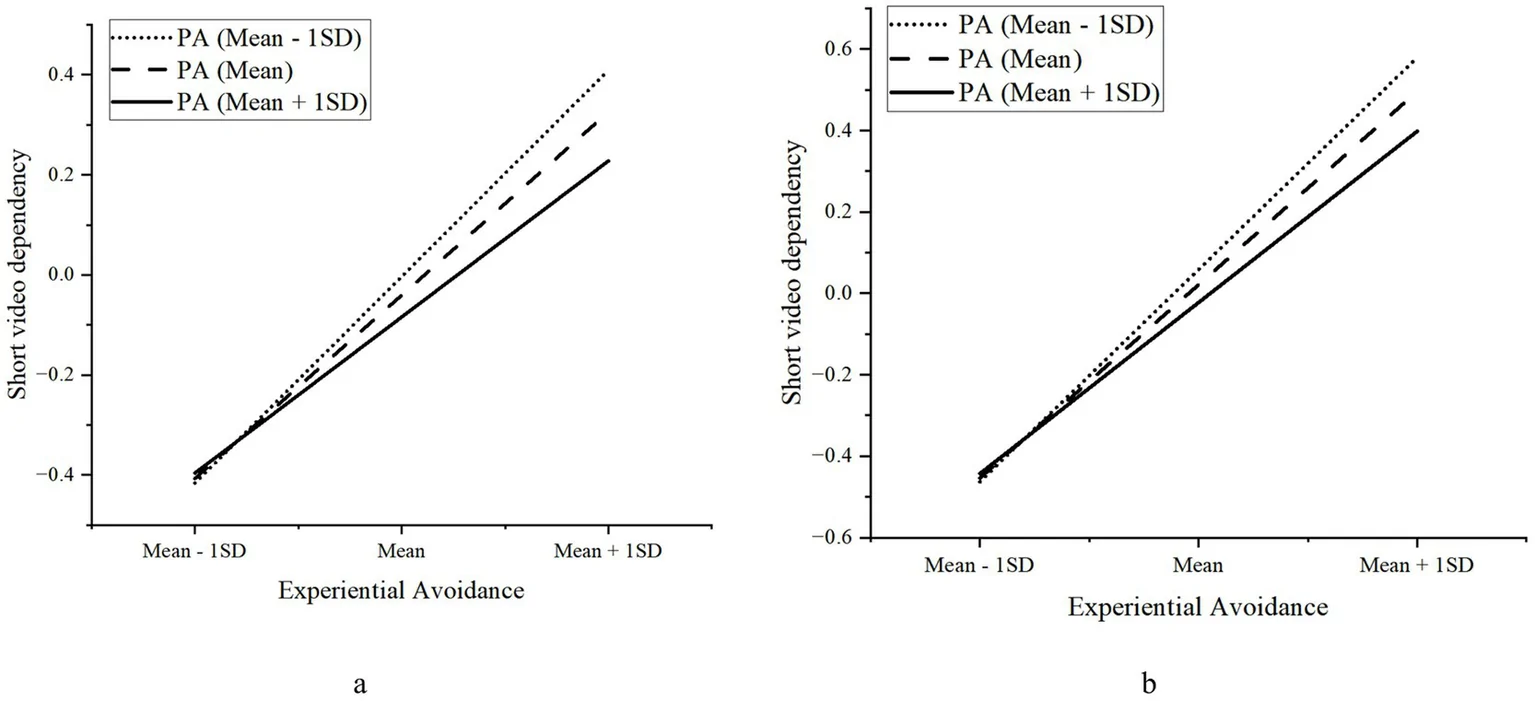
Simple slopes of physical activity in the association between experiential avoidance and short video dependency by gender. **(a)** Boys; **(b)** Girls. PA, physical activity; EA, experiential avoidance; SVD, short video dependency.

**Figure 4:**
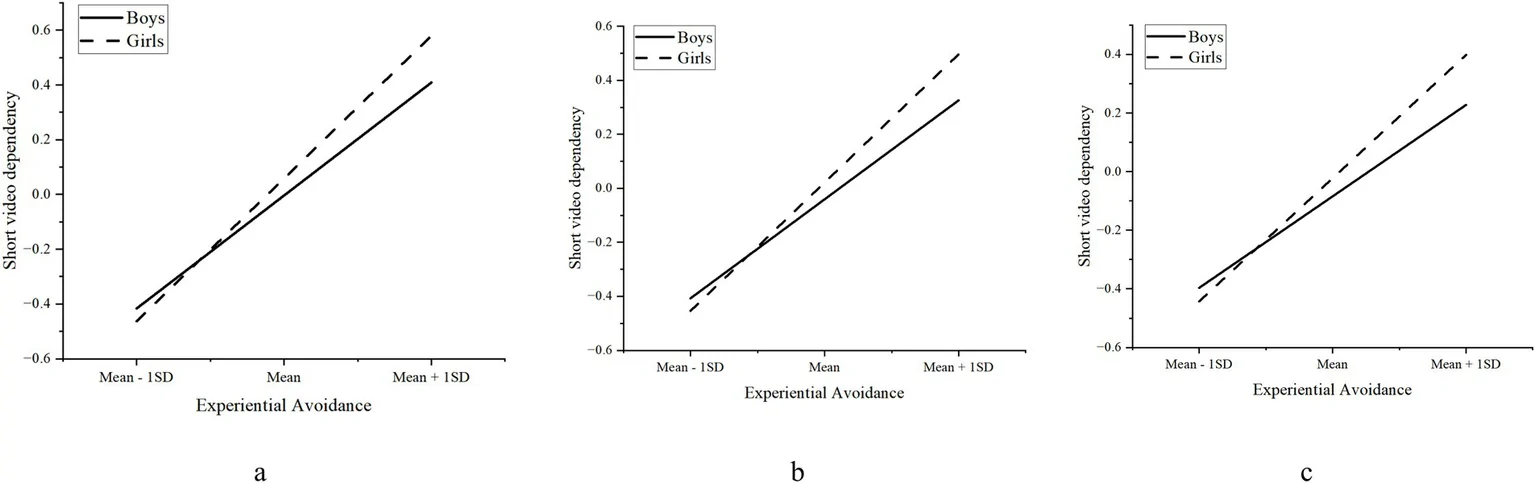
Simple slopes of gender in the association between experiential avoidance and short video dependency across levels of physical activity. **(a)** Low PA (*M* − 1 SD); **(b)** Mean PA; **(c)** High PA (*M* + 1 SD). PA, physical activity; EA, experiential avoidance; SVD, short video dependency; gender was coded as boys = 0 and girls = 1.

After controlling for demographic variables, the results (Table 3; Figures 2–4) indicated that EA was a significant positive predictor of SVD (*β* = 0.4205, *p* < 0.001). Both PA (*β* = −0.0435, *p* < 0.05) and gender (*β* = 0.0310, *p* < 0.05; boys = 0, girls = 1) also exhibited significant main effects. More importantly, the interaction between EA and PA (*β* = −0.0543, *p* < 0.001), as well as the interaction between EA and gender (*β* = 0.0543, *p* < 0.001), were both significant.

**Table 3:** Moderation model predicting short video dependency.

Outcome variable	Predictor variable	*β*	SE	*t*	*R* ^2^	*F*
SVD	EA (*A*)	0.4205	0.0144	29.183***	0.2080	148.4641***
	PA (*B*)	−0.0435	0.0145	−3.0002*		
	*A* × *B*	−0.0543	0.0146	−3.7292***		
	Gender (*C*)	0.0310	0.0144	2.1591*		
	*A* × *C*	0.0543	0.0143	3.8048***		
	Grade	−0.0125	0.0200	−0.6232		
	Age	0.0922	0.0200	4.6077***		

EA, experiential avoidance; PA, physical activity; SVD, short video dependency. Gender was coded as boys = 0 and girls = 1. *p < 0.05; ***p < 0.001.

To further elucidate the moderating role of PA, simple slope analyses were conducted at low (−1 SD), moderate (mean), and high (+1 SD) levels of PA (see Figures 3 and 4). The results demonstrated that at low levels of PA, EA exhibited the strongest positive association with SVD (boys: *β* = 0.4128, *t* = 17.49, *p* < 0.001; girls: *β* = 0.5213, *t* = 22.63, *p* < 0.001). At moderate levels of PA, this predictive effect was attenuated (boys: *β* = 0.3665, *t* = 18.47, *p* < 0.001; girls: *β* = 0.4749, *t* = 22.95, *p* < 0.001). At high levels of PA, the association between EA and SVD was further weakened (boys: *β* = 0.3121, *t* = 12.80, *p* < 0.001; girls: *β* = 0.4206, *t* = 15.88, *p* < 0.001). Overall, as PA levels increased, the positive relationship between EA and SVD progressively diminished, indicating a significant buffering effect of PA.

Gender differences were further examined (Table 4; Figure 4). PA significantly moderated the relationship for both boys and girls, with a stronger buffering effect observed in girls. Specifically, the positive association between EA and SVD was strongest among girls with low PA, whereas it was weakest among boys with high PA.

**Table 4:** Conditional effects of experiential avoidance on short video dependency by physical activity and gender.

Moderating variable (PA)	Moderating variable (gender)	Effect size	SE	*t*	Lower limit 95% CI	Upper limit 95% CI
Low	Boys	0.4128	0.0236	17.4904***	0.3665	0.4591
	Girls	0.5213	0.0230	22.6291***	0.4761	0.5665
Medium	Boys	0.3665	0.0198	18.4736***	0.3276	0.4053
	Girls	0.4749	0.0207	22.9481***	0.4344	0.5155
High	Boys	0.3121	0.0244	12.7972***	0.2643	0.3600
	Girls	0.4206	0.0265	15.8810***	0.3687	0.4726

EA, experiential avoidance; PA, physical activity; SVD, short video dependency. Gender was coded as boys = 0 and girls = 1. ***p < 0.001.

## 4 Discussion

This study examined how EA relates to adolescent SVD and tested whether PA and gender moderate this relationship. Overall, the results supported the three hypotheses. Consistent with H1, EA was positively associated with SVD. Consistent with H2, PA weakened the positive association between EA and SVD, indicating a buffering effect. Consistent with H3, gender moderated this association, with the EA-SVD link being stronger among girls than boys. The study is mainly based on Acceptance and commitment therapy (ACT), which views EA as an unhelpful way to manage negative internal experiences. To further explain how EA may translate into maladaptive media use behaviors and how this pathway may be buffered, the study additionally draws on COR Theory and Self-Regulation Failure Theory. Specifically, EA can be understood as a response to perceived emotional threat, whereas PA may function as a renewable personal resource that helps offset resource depletion. Together, these perspectives provide a coherent framework for understanding the moderating roles of PA and gender in the relationship between EA and SVD.

### 4.1 Experiential avoidance and short video dependency

Consistent with Hypothesis 1, EA was positively associated with SVD. EA represents a maladaptive strategy for coping with negative emotions, manifesting as a tendency to evade difficult tasks or unconsciously withdraw from stress-inducing situations (Hayes et al., 1996). Such avoidance behaviors often drive individuals to seek immediate psychological relief to alleviate emotional distress. Short videos, characterized by immediacy and fragmented content, provide an easily accessible means of escape, particularly for adolescents. Students with high levels of EA are more susceptible to the short-term gratification and sensory stimulation offered by these platforms (Nong et al., 2023b). From the perspective of emotion regulation theory, individuals facing negative emotions often employ external regulation strategies to reduce psychological discomfort (Gross, 2013). Short videos function as an external, instant regulatory tool, enabling attention diversion and temporary relief from unpleasant feelings. Empirical evidence suggests that avoidant individuals are more prone to over-engage with such platforms to escape real-life stressors and negative experiences (Wang Y. et al., 2024). Algorithm-driven content recommendations further reinforce the immersive experience, diminishing motivation to confront real-world problems (Ye et al., 2025). However, these avoidance strategies fail to resolve underlying emotional difficulties and may exacerbate negative experiences, thereby reinforcing dependency (Cheng et al., 2015). These findings align with the Compensatory Internet Use Model (CIUM), which posits that individuals under real-life stress or negative emotional states engage in excessive internet use to achieve compensatory satisfaction (Kardefelt-Winther, 2014b). For adolescents with high EA, the instant entertainment provided by short videos offers a convenient escape to alleviate emotionally challenging circumstances. This mechanism explains the observed positive correlation between EA and SVD. Furthermore, the results can be interpreted through the lens of self-regulation failure theory, which emphasizes the limited nature of self-control resources. Prolonged engagement in emotion suppression and avoidance accelerates depletion of these resources (Molden et al., 2016). Such ego-depletion increases susceptibility to impulsive and uncontrolled behaviors, such as excessive short video consumption for immediate gratification. Consequently, EA not only constitutes a significant risk factor for SVD but may also occupy a central role in the underlying mechanisms driving adolescent media use behaviors.

### 4.2 Moderating role of physical activity

The study found that PA significantly moderated the link between EA and adolescent SVD, supporting Hypothesis 2. According to ACT, EA represents an unhelpful attempt to avoid or manage negative internal experiences. Complementarily, COR Theory suggests that adolescents may rely on available personal resources to maintain psychological stability under stress. In this framework, PA may act as a healthier coping resource that helps adolescents manage unpleasant emotions and reduces their reliance on short videos for immediate relief. Prior research has suggested that regular PA is associated with better emotion regulation, attention control, and impulse inhibition (Loprinzi et al., 2013; Bernstein and McNally, 2018). Therefore, adolescents with higher PA may be less likely to depend on the instant rewards of short videos when experiencing avoidance-related distress. By contrast, adolescents with lower PA may have fewer alternative coping resources and may therefore be more vulnerable to excessive short video use. Because neurobiological mechanisms were not directly measured in this study, these explanations should be interpreted as theoretical possibilities rather than direct evidence. Taken together, the moderation effect of PA suggests that promoting PA may be a useful school-based strategy for reducing the risk associated with EA and SVD.

### 4.3 Moderating role of gender

This study further examined how gender shapes the relationship between EA and SVD, supporting Hypothesis 3. The findings suggest that the positive association between EA and SVD was stronger among girls than among boys. Prior research has shown that females may be more likely to use emotion-oriented coping strategies when facing stress or negative emotions and may show stronger emotional compensation needs in digital media contexts (Tamres et al., 2002; Su et al., 2020). In the present sample, girls also reported lower levels of PA and higher levels of EA and SVD than boys, which provides additional descriptive support for the gender moderation pattern. From a resource-oriented perspective, lower PA may mean fewer opportunities to replenish self-regulatory and emotional resources through activity, which may increase vulnerability when EA is high. At the same time, boys may be more likely to use short videos for leisure or functional purposes rather than primarily for emotional avoidance, which could partly explain the weaker EA-SVD association among boys (Liang et al., 2016). Some previous studies have discussed possible sex-related differences in exercise-related executive functioning (Barha et al., 2017), but the present study did not directly measure neurophysiological processes; therefore, this interpretation should remain cautious. Overall, gender appears to be an important boundary condition in the EA-SVD pathway, suggesting that future interventions should consider gender-sensitive strategies, particularly by increasing accessible PA opportunities and emotion regulation support for girls with high EA.

### 4.4 Research significance

This study examined how EA relates to adolescent SVD and tested whether PA and gender influence this relationship. The results provide new insights into individual differences in media use behaviors. PA was found to significantly buffer the negative impact of EA on SVD, with this moderating effect differing across genders, suggesting that gender may play a meaningful explanatory role in this pathway. From an integrated theoretical perspective, the findings demonstrate that ACT explains EA as a core psychological vulnerability, while COR Theory clarifies how PA functions as a buffering resource against emotional depletion. The emotion regulation model and Self-Regulation Failure Theory further elucidate how deficits in emotional control and impulse inhibition translate EA into excessive short video use. Practically, the findings suggest several avenues for targeted intervention by schools and health educators. First, integrating regular aerobic or group physical activities into daily school curricula may enhance students’ self-regulation abilities and reduce tendencies toward EA. Second, interventions should account for gender differences in media literacy education—for instance, enhancing self-control and impulse management for male students while providing female students with greater access to emotion regulation and psychological support resources. Third, mindfulness training and workshops can cultivate emotional expression and coping skills, reducing the adverse impact of EA on SVD. Overall, this study emphasizes the importance of considering both behavioral characteristics and demographic variables when understanding and intervening in SVD. Future research could adopt interdisciplinary perspectives, including developmental psychology, gender studies, and health behavior, to further investigate the dynamic mechanisms of these moderation pathways and validate their applicability across cultures and developmental stages.

### 4.5 Strengths and limitations

This study contributes theoretically by systematically examining how PA and gender moderate the relationship between EA and SVD, enriching our understanding of media dependency formation pathways and highlighting the joint influence of individual behavioral factors and demographic variables. Methodologically, the inclusion of interaction term analyses allowed identification of heterogeneity in moderating effects across different groups, demonstrating empirical innovation. Moreover, the focus on adolescents provides practical relevance, offering guidance for optimizing media behavior management and promoting healthy lifestyles.

Despite its contributions to elucidating the association between EA and adolescent SVD and the underlying moderating mechanisms, the present study has several limitations that should be acknowledged. First, this study employed a cross-sectional design, which precludes definitive conclusions regarding temporal ordering and causal direction among the variables. In particular, the relationship between EA and SVD may reflect a unidirectional pathway in which psychological vulnerability predisposes adolescents to addictive media use, or a reciprocal process whereby excessive short video consumption reinforces avoidance-oriented coping tendencies. Future research should adopt longitudinal designs, cross-lagged panel models, or experimental intervention approaches to disentangle these dynamic pathways more rigorously. Second, the study relied primarily on self-report questionnaires. Although this approach offers practical advantages in large-scale data collection, behavioral variables such as PA and short video use may be subject to recall bias and social desirability effects. To enhance ecological validity and measurement accuracy, future studies are encouraged to integrate objective assessment methods, such as ecological momentary assessment (EMA), wearable devices, or digital usage logs. Third, the brief PA scale used in this study demonstrated relatively limited internal consistency. While this scale is better understood as a composite behavioral index than as a traditional psychological scale, its reduced reliability may lead to conservative estimates of moderation effects. Fourth, several mechanistic explanations discussed in this study, including emotion regulation and possible neurocognitive pathways, were not directly measured. Future studies should include direct measures of these mechanisms to test the proposed explanations more rigorously.

## 5 Conclusion

This study used an adolescent sample to investigate how EA is associated with SVD, focusing on the moderating effects of PA and gender. The findings revealed a significant positive relationship between EA and SVD, indicating that adolescents with higher EA may be more vulnerable to short video dependency. PA weakened this positive association, suggesting a potential protective role. Gender also moderated the relationship, with the EA-SVD association being stronger among girls than boys. These findings suggest that interventions aimed at reducing adolescent SVD should consider both behavioral resources, such as PA, and gender-sensitive prevention strategies. Given the cross-sectional design, these conclusions should be interpreted as evidence of associations rather than causal effects.

## Data Availability

The datasets generated and/or analyzed during the current study are not publicly available due to privacy and ethical restrictions related to the involvement of adolescent participants, but are available from the corresponding author on reasonable request.
