# Supplementary material for: A dual-moderation model of physical activity and gender in the relationship between experiential avoidance and short video dependency
Source: Front Psychol. 2026 Aug 31;17:1757041. doi: 10.3389/fpsyg.2026.1757041 (PMC13572727; doi:10.3389/fpsyg.2026.1757041)
Supplement: Supplementary file 1 [file Data_Sheet_1.PDF]

1 = Strongly disagree 2 = Slightly disagree 3 = Not sure 4 = Agree 5 = Strongly agree

Question number	Content	1	2	3	4	5
1	Due to excessive use of short video media, I have become indifferent to the outside world and interacted with it less.					
2	Once I open a short-video app, I find it hard to quit immediately. I always want to watch a little longer to feel satisfied.					
3	Short videos have accustomed me to obtaining a large amount of information in a short period of time. I feel that my patience has deteriorated.					
4	Frequent or prolonged viewing of short videos has caused me to often suffer from insufficient sleep or poor sleep quality.					
5	Constantly watching short videos has made me lose interest in and develop resistance towards longer activities.					
6	Watching short videos often makes me sit for long periods and lack exercise, thus causing me to feel tired frequently.					
7	Frequent or prolonged viewing of short videos often causes me to experience dry eyes and visual fatigue.					
8	Watching short videos for a long time has reduced the time I have for physical activities.					
9	Watching short videos for a long time in a fixed position caused my fingers or cervical vertebrae to feel sore.					
10	I recommend short videos I like or find interesting to my friends					
11	I think it's more convenient and efficient to obtain information through short videos.					
12	I have received short videos shared by my friends.					
13	Through the short-video network, I can promptly obtain the information of the people I follow.					

1=非常不符合；2=不太符合；3=不确定；4=比较符合；5=非常符合

题号	内容	1	2	3	4	5
1	由于过度使用短视频媒体，使得我对外界漠不关心，与外界的互动变少					
2	我只要打开短视频 APP 难以立刻退出来，总想多看一会才满足					
3	短视频让我习惯了短时间内获取大量信息，我感觉自己的耐心变差了					
4	频繁或长时间观看短视频，使我经常睡眠不足或睡眠质量差					
5	经常观看短视频，使我对时间较长的活动提不起兴趣或产生抗拒情绪					
6	观看短视频使我常常久坐，缺乏运动，因而经常感到疲惫					
7	频繁或长时间观看短视频，使我时常感到眼睛干涩、视觉疲劳					

8	长时间观看短视频使我进行身体活动的时间变少了					
9	长时间固定姿势观看短视频，使我的手指或颈椎感到酸痛					
10	我会把我喜欢的、有趣的短视频推荐给好友					
11	我觉得从短视频获取信息更为便捷、高效					
12	我有收到过好友分享给我的短视频					
13	通过短视频网络，我可以及时获取关注的人的信息					
